# Effects of Male Hypogonadism on Regional Adipose Tissue Fatty Acid Storage and Lipogenic Proteins

**DOI:** 10.1371/journal.pone.0031473

**Published:** 2012-02-20

**Authors:** Sylvia Santosa, Michael D. Jensen

**Affiliations:** 1 Endocrine Research Unit, Mayo Clinic, Rochester, Minnesota, United States of America; 2 Department of Exercise Science, Concordia University, Montreal, Canada; University of Las Palmas de Gran Canaria, Spain

## Abstract

Testosterone has long been known to affect body fat distribution, although the underlying mechanisms remain elusive. We investigated the effects of chronic hypogonadism in men on adipose tissue fatty acid (FA) storage and FA storage factors. Twelve men with chronic hypogonadism and 13 control men matched for age and body composition: 1) underwent measures of body composition with dual energy x-ray absorptiometry and an abdominal CT scan; 2) consumed an experimental meal containing [^3^H]triolein to determine the fate of meal FA (biopsy-measured adipose storage vs. oxidation); 3) received infusions of [U-^13^C]palmitate and [1-^14^C]palmitate to measure rates of direct free (F)FA storage (adipose biopsies). Adipose tissue lipoprotein lipase, acyl-CoA synthetase (ACS), and diacylglycerol acetyl-transferase (DGAT) activities, as well as, CD36 content were measured to understand the mechanism by which alterations in fat storage occur in response to testosterone deficiency. Results of the study showed that hypogonadal men stored a greater proportion of both dietary FA and FFA in lower body subcutaneous fat than did eugonadal men (both p<0.05). Femoral adipose tissue ACS activity was significantly greater in hypogonadal than eugonadal men, whereas CD36 and DGAT were not different between the two groups. The relationships between these proteins and FA storage varied somewhat between the two groups. We conclude that chronic effects of testosterone deficiency has effects on leg adipose tissue ACS activity which may relate to greater lower body FA storage. These results provide further insight into the role of androgens in body fat distribution and adipose tissue metabolism in humans.

## Introduction

The role of testosterone in the sexual dimorphism of body composition is most obvious in conditions of changing or abnormal testosterone levels in males. The amount of central vs. extremity adipose tissue, as measured by the ratio of trunk to peripheral skin fold thicknesses, begins to increase during male puberty and continues to do so into adulthood [1]. Men with chronic testosterone deficiency, such as Klinefelter's syndrome or male eunuchs, have a more feminine/gynoid pattern fat distribution [2], [3]. In contrast, testosterone administration to men over 65 years old with somewhat low serum testosterone concentrations decreased fat mass principally in the arms and legs [4]. How testosterone affects body fat patterning is of interest because an upper body fat distribution in obese individuals increases disease risk relative to those with a predominantly lower body fat distribution. The mechanism by which testosterone causes fat to be stored preferentially in some depots is presently unknown. In this study we investigated the effects of chronic testosterone deficiency on fatty acid (FA) metabolism by comparing a group of hypogonadal men to a group of eugonadal men.

Fatty acids stored in adipose tissue largely derive from triglyceride rich lipoproteins (chylomicron and VLDL). A portion of fatty acids are redistributed between depots from the circulating free (F)FA pool that we've referred to as the direct FFA storage pathway. Dietary FA in chylomicrons require lipoprotein lipase (LPL) in order to be taken up by adipocytes and testosterone treatment of hypogonadal men decreases LPL activity [5]–[7]. However, nothing is known about testosterone's role in regulating direct FFA storage in adipocytes or the major steps that regulate adipocyte fatty acid processing. We have reported that differences in direct FFA storage in men and women are consistent with body fat patterning, suggesting this pathway may at least in part regulate body fat distribution [8]. In this study we examined the effects of chronic hypogonadism on both meal-derived FA metabolism and the direct FFA storage pathway.

Fatty acids can enter adipocytes through passive (flip-flop) or protein facilitated diffusion mechanisms [9]. Once inside the cell, they must undergo a series of enzymatic reactions to be stored as triglyceride. Herein we report the effects of chronic testosterone deficiency on the activity of some key factors involved in the storage of FA as triglyceride. The acyl CoA synthetase enzymes (ACS), diacylglycerol acyltransferase enzymes (DGAT), and fatty acid transport protein (CD36) are factors involved in three different tiers of adipocyte FA storage. CD36 is a ubiquitously expressed cell-surface glycoprotein that is abundant on adipocyte plasma membranes [10] and is implicated in membrane binding and transport facilitation of FA into the cell [11]. ACS enzymes catalyze the activation of FA to long chain acyl-CoA [12]. Lastly, DGAT catalyzes the final step of fatty acid storage as triglycerides by esterifying a fatty acid to a diglyceride (diacylglycerol) molecule [13], [14]. In this study we find unique differences between men with and without chronic testosterone deficiency/hypogonadism in adipose tissue FA storage factors. In addition, we find variations in the relationships between these proteins and FA storage that may explain how body fat patterning is regulated by testosterone status.

## Methods

### Subjects

We recruited 12 men who had undergone androgen deprivation treatment therapy for at least 6 months to prevent prostate cancer recurrence. For the purposes of this report we will refer to this group as testosterone deficient or T(−). Only men with normal prostate specific antigen (PSA) concentrations and no evidence of prostate cancer were included. Thirteen men with normal serum testosterone concentrations, or T(+), were recruited as age- and BMI-matched controls. All participants were weight stable, defined as ±1.0 kg, for >2 months before the study and were not taking other medications that could affect FA metabolism. Prior to study participation all men underwent a physical examination and clinical blood tests to ensure they were healthy and free of disease. Written, informed consent was obtained from all participants. The study was approved by the Institutional Review Board of the Mayo Clinic.

### Materials

[l-^14^C]palmitate, and [9,10-^3^H]triolein were purchased from NEN Life Science Products (PerkinElmer, Boston, MA). [U-^13^C]palmitate and ^2^H_2_O were purchased from Isotec (99 atom %, Miamisburg, OH).

### Study Design

All studies were conducted in the Mayo Clinical Research Unit (CRU). Body composition was measured prior to the inpatient study. To ensure all participants were in comparable nutritional states they received meals from the CRU Metabolic Kitchen for 5 days prior to the inpatient study days. Macronutrient content of the diet was 45% carbohydrate, 35% fat and 20% protein. On the evening of the fifth day of the dietary control period participants were admitted to the CRU for their inpatient study. The following morning they consumed an experimental meal containing [^3^H]triolein as previously described [15]. Blood samples were collected just prior to and hourly after the meal for 10 hours. A 24 h urine collection was performed to measure nitrogen and ^3^H_2_O excretion. Energy expenditure and respiratory exchange ratios (RER) were measured hourly for 6 hours using indirect calorimetry (DeltaTrac, Yorba Linda, CA). Abdominal and femoral adipose tissue biopsies were performed at 1400 h for measurement of “fed” LPL activity. After a second consecutive overnight visit, FFA tracers were given to measure direct FFA storage. A second set of adipose tissue biopsies were performed on the contra-lateral side 30 minutes after the bolus infusion of [1-^14^C]palmitate (see below).

### Assays and Methods

Chylomicron particles were separated by ultracentrifugation of 0.8 mL of fresh plasma as previously described [16]. The triglyceride concentrations in both the chylomicron and non-chylomicron fractions were measured and the remainder of the plasma from these fractions was extracted using a Dole solution and assayed for ^3^H content. Urinary nitrogen was measured using an Analox GM7 Fast Enzymatic Metabolite Analyzer (Analox Instruments, Lunenburg, MA). Plasma glucose was measured using a glucose analyzer (Beckman Instruments, Fullerton, CA) and plasma catecholamine concentrations in plasma were measured by reversed phase HPLC [17]. Plasma triglyceride concentrations were measured using a microfluorometric assay [18]. Plasma insulin concentrations were measured using chemiluminescent assays on an automated immunoassay system (Assay and DxI, Beckman Instruments, Chaska, MN). Plasma estrogen and testosterone were measured via LC/MS.

#### Body composition

Dual-energy x-ray absorptiometry (DXA) (Lunar iDXA, GE Healthcare, Madison, WI) was used to measure fat-free mass (FFM), total body mass, and leg fat mass (FM) [19]. Abdominal subcutaneous and visceral adipose tissue areas were determined using a single-slice abdominal computed tomography (CT) scan at the L2–L3 level. The CT data, combined with the DXA-measured total abdominal fat content, were used to calculate visceral fat mass [20]. Total body water was measured as per Schoeller et al [21].

#### Substrate oxidation

Participants fasted for 12 hours prior to measuring resting energy expenditure (REE). Overnight postabsorptive rates of oxygen consumption (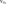) and carbon dioxide production (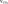) were measured for 30 minutes with a 5 minute acclimatization period. Thereafter, measurements were taken every hour for 15 minutes from 0 to 360 minutes after the consumption of the meal labeled with [^3^H]triolein. Carbohydrate and fat oxidation at each time point were determined [22] and 6 hour fat and carbohydrate oxidation (g) was calculated using a trapezoidal approach.

#### Meal fatty acid storage and oxidation

At 0800 h on the morning after admission participants consumed a liquid meal (Ensure Plus, Ross Laboratories, Abbott, Columbus, OH) that provided energy equivalent to 40% of their individually measured REE. Ensure Plus is 57% carbohydrate, 27% fat, and 15% protein. The experimental meal was labeled with 50 µCi [9,10-^3^H]triolein as previously described [15]. Quadruplicate 50-µL samples of the test meal were counted on a liquid scintillation counter to determine the exact amount of [^3^H]triolein consumed. Lunch and supper were provided at 1300 and 1800 h and consisted of solid foods with the same macronutrient composition as those provided during the week before admission. During the first 8 hours after the test meal, the participants remained in bed except as needed to void. In order to measure the ^3^H_2_O concentration in total body water we collected a fresh urine sample (shortly after completely voiding) 24 h after consuming the [^3^H]triolein-labeled meal. An aliquot of the 24 h urine collection and the freshly voided sample were analyzed for ^3^H_2_O concentration.

#### Direct adipose tissue FFA storage

At 0700 h the second morning of the study a continuous infusion of [U-^13^C]palmitate was started at a rate of 2 nmol•kg^−1^•min^−1^ to measure FFA flux [23]. At 0800 h a bolus infusion of [1-^14^C]palmitate was given to measure direct adipose tissue FFA storage [8]. We were unable to collect valid palmitate flux data in one control volunteer because of problems with the intravenous infusion line for the [U-^13^C]palmitate. Thus, for palmitate flux and rates of adipose tissue palmitate storage n = 12 for control men.

Abdominal and femoral adipose tissue biopsies were obtained using sterile technique and local anesthesia 6 and 24 hours after the experimental meal. The 24 h biopsy corresponded to the 30 minute post-bolus time point to allow the simultaneous measurement of direct FFA storage and 24 h meal fatty acid storage. By collecting adipose tissue biopsies at 30 minutes post-bolus we minimize the appearance of FFA tracer into VLDL particles while simultaneously assuring virtually 100% clearance of the palmitate tracer from the circulation [8]. Adipocytes were isolated using collagenase digestion and the adipocyte lipids were extracted using the Folch method [24]. Adipocyte ^3^H and ^14^C lipid specific activity (SA, dpm·g lipid^−1^) were measured as previously described [25]. Extracted lipids were weighed and counted on a scintillation counter to <2% counting error.

#### Adipose tissue analysis

Fat cell size was measured using photomicrographs [26]. Adipose tissue heparin-releasable LPL activity was measured using the approach of Nilsson-Ehle and Schotz [27]. To measure CD36 and ACS activity adipose tissue is homogenized and the lipid cake separated from cellular components (whole tissue extract). For DGAT activity the whole tissue extract is further purified to remove membrane proteins and the assay is performed on the remaining cytosolic fraction. ACS [28] and DGAT [14] activity were determined using enzymatic assays and CD36 quantification was measured using sandwich ELISA [29].

### Calculations

Regional meal FA storage and oxidation (percent and mass units) were calculated as previously described [30]. Palmitate flux, fractional direct FFA storage, regional FFA storage rates as determined by ^14^C administration were calculated as previously described [31]. Regional tissue FFA storage rates (µmol•kg adipose tissue^−1^•min^−1^) were calculated as the product of regional FFA storage rate and flux [31]. The regional FA storage rates per unit tissue are determined by the activity/behavior per cell and the number of cells. To understand how factors operating at the level of the adipocyte contribute to FA storage at the unit of tissue mass, we also present cell FFA storage rates (µmol•1000 cells^−1^•min^−1^), which is calculated as the product of regional tissue FFA storage rate and fat cell size divided by 1000 [31].

### Data Analysis and Statistics

The means of data expression can impact data interpretation [32]. Herein we use the per unit lipid expression when the question relates to whether one body fat depot competes better for the available fatty acids than other depots. However, when examining the factors that regulate tissue FA storage rates at a cellular level we express the data per 1000 adipocytes. All data were tested for normality using the Shapiro-Wilk test for goodness of fit. Data that were not normally distributed were log transformed. Between group comparisons were assessed by unpaired t-tests. A mixed model ANOVA was used to determine between and within group differences using time and depot as within group variables. Post hoc tests were conducted using least squares means contrast tests. Rather than doing partial analyses using log transformed data, correlations in T(−) and T(+) groups were determined using Spearman's rank test. All data are presented as mean ± SEM and were analyzed using JMP 8.0 (SAS Institute, Cary, NC). Statistical significance was defined as p<0.05.

## Results

### Subject Characteristics

Participants were intentionally matched for age, BMI, and body composition (Table 1). The range of BMI's observed in the T(−) group was 23.9–40.6 kg/m^2^, and thus we recruited a control group with a similar range (23.8–39.1 kg/m^2^) and average BMI. By design, total and regional fat mass were not significantly different between the two groups, serum testosterone concentrations were undetectable in the T(−) group and normal in the T(+) group. As expected, serum estrogen concentrations were greater in T(+) than T(−) men. Femoral adipocytes were larger (p = 0.005) than abdominal adipocytes in T(+), but not T(−) men.

**Table 1:** Subject characteristics.

	Normal Testosterone (n = 13)	Low Testosterone (n = 12)
Age	56±1	57±2
Weight (*kg*)	97.0±4.9	93.4±4.6
BMI (*kg/m^2^*)	29.8±1.2	30.1±1.3
Fat (*%*)	33±2	35±2
Fat (*kg*)	31.7±3.5	32.2±3.1
Upper body SQ (*kg*)	16.3±2.1	17.2±1.9
Lower body SQ (*kg*)	8.8±0.8	9.9±0.7
Visceral fat (*kg*)	6.7±1.0	5.0±0.8
Abdominal fat cell size (*µg lipid·cell^−1^*)	0.71±0.07*	0.84±0.07
Femoral fat cell size (*µg lipid·cell^−1^*)	0.92±0.11	0.86±0.08
Testosterone (*ng·dL^−1^*)	287±19	<50
Estradiol (*pg·mL^−1^*)	17.6±1.3	3.3±0.3†
Fasting insulin (*µU·L^−1^*)	8.9±2.1	7.7±1.7
Fasting plasma glucose (*mg·dL^−1^*)	100±2	102±3
QUICKI	0.36±0.01	0.36±0.01
Plasma palmitate (*µmol·L^−1^*)	92±4	93±7
Palmitate flux (*µmol·min^−1^*)	117±8	106±12

Values are mean ± SEM.

*p<0.05 between upper and lower body within groups.

† p<0.05 between groups. Testosterone concentrations were not random variables and therefore not subject to statistical testing. For palmitate flux n = 12 for the normal testosterone group.

**Figure 1:**
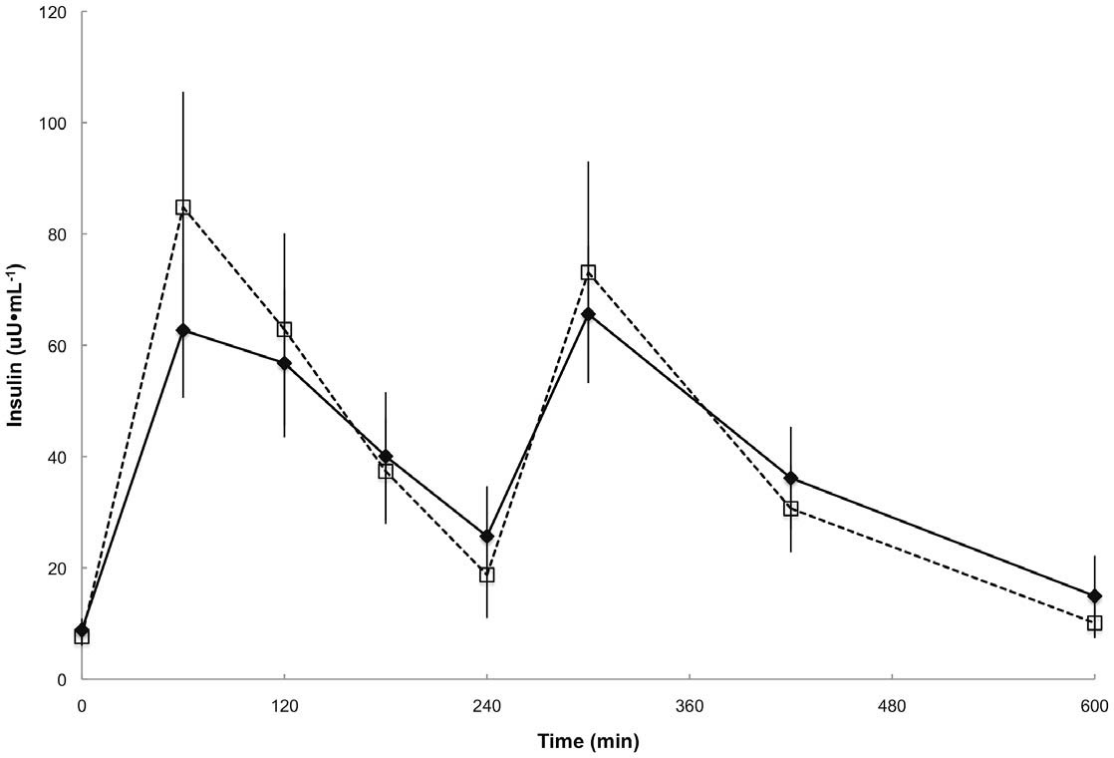
Daytime plasma insulin concentrations. Plasma insulin concentrations during the first 10 h of the experimental meal day. ♦,**—**represents T(+) men, □, **- - -**represents T(−) men. There were no significant differences between groups at any time point.

Plasma insulin concentrations were similar between groups throughout the meal study day (Figure 1). Plasma epinephrine and norepinephrine concentrations were not different between groups at baseline and before the biopsy (data not shown). The average overnight postabsorptive plasma palmitate concentrations and flux were not different in the two groups (Table 1).

**Figure 2:**
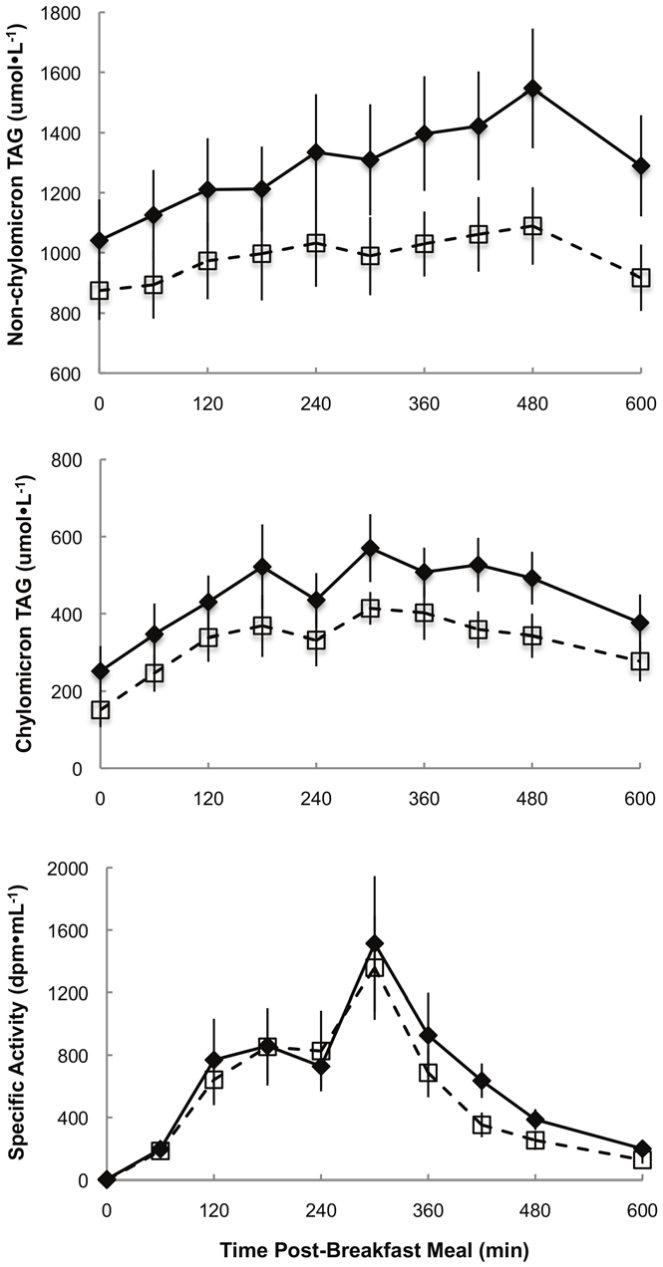
Daytime plasma triglyceride concentrations and specific activity. Plasma non-chylomicron (top panel) and chylomicron triglyceride concentrations (middle panel) and chylomicron triglyceride specific activity (lower panel) during the first 10 h of the experimental meal day. ♦,**—**represents T(+) men, □, **- - -**represents T(−) men. There were no differences between groups across time for chylomicron triglyceride concentrations and SA. Non-chylomicron triglycerides were greater (p = 0.02) in T(+) men across time.

Non-chylomicron triglyceride concentrations were greater (p = 0.02) in T(+) than T(−) men (Figure 2). However, there were no significant between-group differences in chylomicron triglyceride concentrations or SA, suggesting that meal FA absorption/transport into the circulation was similar in the two groups.

### Substrate and Meal FA Oxidation

There were no differences in overnight postabsorptive REE and respiratory exchange ratio (RER) between groups (Table 2) or between inpatient study days, indicating that the participants were in a metabolically steady state. Indirect calorimetry measures of post-breakfast substrate oxidation revealed that T(+) men oxidized more fatty acids (p = 0.04) over 6 hours than did T(−) men (Table 2). However, the oxidation of meal fatty acids (^3^H_2_O generation) over 24 hours was not significantly different between groups.

**Table 2:** Energy and fatty acid oxidation.

	Normal Testosterone (n = 13)	Low Testosterone (n = 12)
REE (*kcal·d^−1^*)	2052±83	1846±87
Respiratory Exchange Ratio	0.79±0.01	0.80±0.01
6 h substrate oxidation:		
Carbohydrate (*g*)	87±1	90±7
Fat (*g*)	32±2^x^	25±3^y^
Protein (*g*)	26±1	26±1
Meal fatty acid oxidation:		
(*g*)	11.5±0.9	9.6±0.7
(*%*)	46±3	42±2

Values are mean ± SEM. REE – resting energy expenditure. 6 h substrate oxidation refers to the indirect calorimetry data collected just before and for 6 h following the experimental breakfast meal. Meal fatty oxidation refers to isotope measures of ^3^H-triolein disposal into adipose tissue (as assessed by biopsies and generation of ^3^H_2_O) 24 h after consumption of the experimental breakfast meal.

x,y p<0.05 between groups (ie. T(+) vs. T(−)).

### Regional Meal FA Storage

Although average breakfast meal fatty acid storage (mg meal FA·g lipid^−1^) was not significantly different between groups, the proportion of meal FA stored in lower body subcutaneous adipose tissue (LBSQ) was greater (p = 0.02) in T(−) than T(+) men (Table 3). Average breakfast meal fatty acid storage (mg meal FA·g lipid^−1^) was identical in abdominal and femoral fat in T(−) men and slightly, but not significantly greater in abdominal than femoral fat in T(+) men. Because men had more upper than lower body fat, the proportion of meal FA stored in upper body subcutaneous adipose tissue (UBSQ) was greater than in the LBSQ for both groups (p<0.001 for both).

**Table 3:** Fatty acid storage.

	Normal Testosterone (n = 13)		Low Testosterone (n = 12)	
	Abdominal	Femoral	Abdominal	Femoral
Meal fatty acid storage (*mg·g lipid^−1^*)	0.22±0.04	0.18±0.04	0.21±0.02	0.21±0.01
Rate of direct FFA storage:				
(*µmol•kg adipose tissue^−1^•min^−1^*)	0.19±0.03	0.18±0.02	0.18±0.03	0.20±0.03
×10^−4^ (*pmol•1000 cells^−1^•min^−1^*)	1.28±0.22	1.72±0.36	1.60±0.39	1.89±0.43
Adipose tissue fatty acid storage (*%*):				
	UBSQ	LBSQ	UBSQ	LBSQ
Meal fatty acid	13±2^a^	7±2^b^^,^ x	16±2^a^	10±1^b^^,^ y
Direct FFA	2.3±0.3^a^	1.3±0.2^b^^,^ x	2.9±0.5^a^	1.9±0.2^b^^,^ y

Values are mean ± SEM. Meal fatty acid storage refers to isotope measures of ^3^H-triolein disposal into adipose tissue (as assessed by biopsies) 24 h after consumption of the experimental breakfast meal. Direct adipose FFA storage rates are based on isotope dilution measures using adipose biopsies and palmitate kinetics.

a,b p<0.05 between depots within a group;

x,y p<0.05 between groups (ie. T(+) vs. T(−)).

### Direct Adipocyte FFA Storage

There were no statistically significant differences in FFA-palmitate storage rates between groups or depots (Table 3). The proportion of direct FFA storage was greater in UBSQ than in LBSQ in both T(−) and T(+) men (p = 0.003, p = 0.002, respectively). The proportion of direct FFA storage in LBSQ was greater (p = 0.04) in T(−) than T(+) men.

### Adipose Specific Effectors of FA Storage

*LPL activity*. Fasting LPL activity was greater in femoral than abdominal adipose tissue (p = 0.002 and p = 0.01 for T(−) and T(+) men, respectively) (Table 4). Abdominal LPL activity was greater in the fed than fasted state in T(−) (p = 0.004) and T(+) (p = 0.03) men, whereas femoral LPL activity was not significantly different between the fasted vs. fed condition in either group.

**Table 4:** Adipose tissue characteristics.

	Normal Testosterone(n = 13)		Low Testosterone(n = 12)	
	Abdominal	Femoral	Abdominal	Femoral
LPL activity (*µmol•g tissue^−1^•h^−1^*):				
Fasted	0.71±0.06^a^^,^ n	1.38±0.47^b^	1.10±0.20^a^^,^ n	2.20±0.57^b^
Fed	1.12±0.17^m^	1.44±0.25	1.90±0.41^m^	1.91±0.31
Adipocyte factors (•*mg lipid^−1^*):				
ACS (*pmol*•*min^−1^*)	54.3±10.4	46.5±3.9^x^	48.6±3.5^a^	72.0±6.6^b^^,^ y
DGAT (*pmol*•*min^−1^*)	5.7±1.0^a^	3.9±0.3^b^	5.3±0.5	4.7±0.3
CD36 (*relative units*)	19.7±2.2	19.5±3.1	21.4±3.6	21.0±3.1
Adipocyte factors (•*1000 cells^−1^*):				
ACS (*pmol*•*min^−1^*)	31.9±2.9^a^	43.1±6.3^b^^,^ x	41.4±4.7^a^	60.9±7.2^b^^,^ y
DGAT (*pmol*•*min^−1^*)	3.4±0.2	3.6±0.5	4.5±0.6	3.9±0.4
CD36 (*relative unit*)	12.6±1.2	17.0±2.5	18.7±4.0	18.2±3.7

Values are mean ± SEM.

a,b p<0.05 between depots within a group.

m,n p<0.05 across time (ie. fasted vs. fed).

x,y p<0.05 between groups (ie. T(+) vs. T(−)).

#### LPL activity vs. Meal FA storage

**Figure 3:**
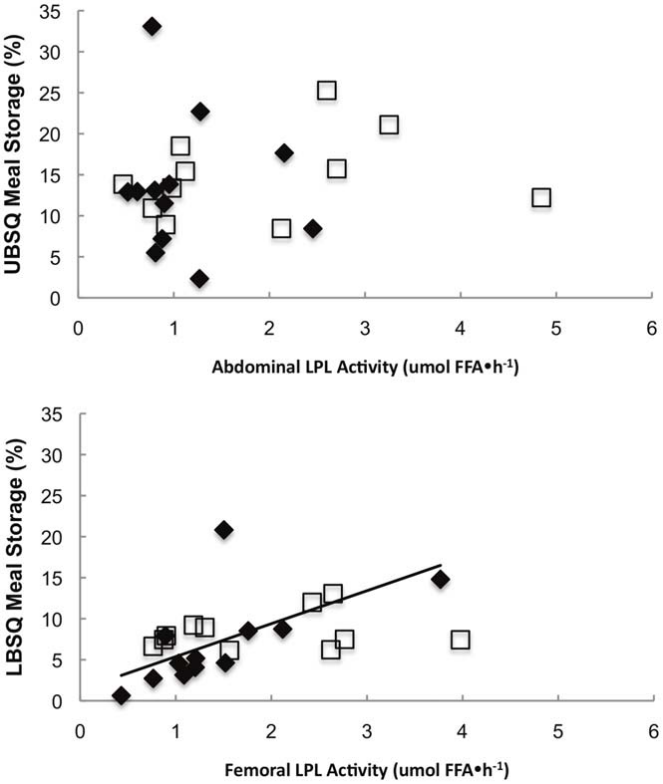
Regional LPL activity vs. regional meal fatty acid storage. Fed LPL activity in the abdominal (top panel) and femoral (lower panel) is plotted vs. the proportion of meal fatty acids stored in upper body subcutaneous (UBSQ) and lower body subctuaneous (LBSQ) adipose tissue. ♦represents T(+) men, □represents T(−) men, — represents a significant correlation in T(+) men.

Because fed LPL activity is a predictor of fractional, regional meal FA storage [33] in young, lean healthy men and women, we examined whether fed LPL activity correlated with the proportion of meal fat stored in upper and lower body subcutaneous depots in this population. Indeed, femoral LPL activity correlated positively with the proportion of meal FA stored in LBSQ in T(+) men (ρ = 0.77, p = 0.004), but this was not the case in T(−) men (Figure 3). Fed LPL activity in abdominal subcutaneous adipose tissue was not correlated with fractional UBSQ meal FA storage in either group. Meal fat storage in femoral fat expressed as mg meal FA stored⋅g adipose tissue lipid^−1^ was correlated with fed femoral LPL activity in both T(−) and T(+) men (ρ = 0.65, p = 0.03; ρ = 0.73, p = 0.007, respectively) (Figure 3). Abdominal meal FA storage (mg⋅g adipose tissue lipid^−1^) in T(+) and T(−) men was not associated with fed or fasted LPL activity.

#### Adipocyte FA storage factors

Table 4 provides overnight postabsorptive adipose tissue CD36 content, ACS and DGAT activity data expressed at both a physiological (per mg lipid) and cellular (per 1000 cell) level. Regardless of whether ACS activity is examined at the physiological or cellular level, T(−) men had greater (pmol•mg lipid^−1^•min^−1^, p = 0.01; pmol•1000 cells^−1^•min^−1^, p = 0.03) femoral ACS activity than T(+) men indicating a clear upregulation of activity in T(−) men. In T(−) men, there were consistent between depot differences in ACS activity, with femoral being greater than abdominal (pmol•mg lipid^−1^•min^−1^, p = 0.004; pmol•1000 cells^−1^•min^−1^, p = 0.047). In T(+) men, ACS activity was greater (p = 0.003) in femoral than abdominal adipose tissue when expressed per 1000 cells, but not per mg lipid.

DGAT activity (pmol•mg lipid^−1^•min^−1^) was greater (p = 0.02) in abdominal than femoral adipose tissue in T(+) men, but not T(−) men. When scrutinized at the cellular level (per 1000 cells), DGAT activity in T(+) men was no longer different. The physiological but not cellular difference in DGAT activity between depots in T(+) men may be explained by smaller abdominal vs. femoral fat cell size in T(+) men (Table 1). These differences mean that the greater DGAT activity in the abdominal fat mass of T(+) men relates primarily to a greater number of smaller adipocytes. There were no significant differences in adipose tissue CD36 content between depots or groups.

#### Adipocyte proteins vs. Direct FFA storage rates

**Figure 4:**
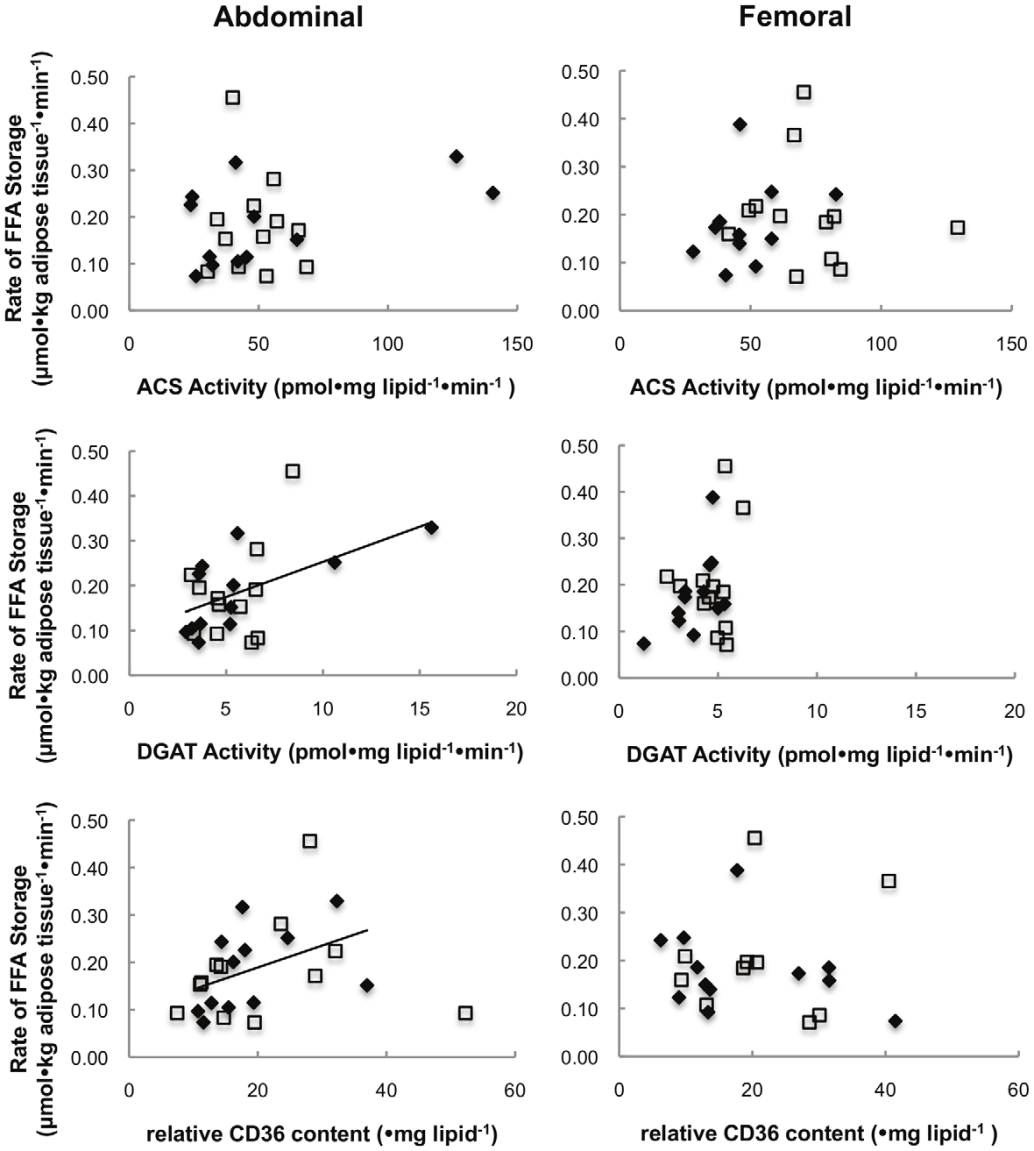
Physiological relationship between regional FA storage factors vs. regional FFA storage. Regional rates of FFA storage (µmol•kg adipose tissue^−1^•min^−1^) vs. lipogenic protein activity expressed per mg lipid. ♦represents T(+) men, □represents T(−) men, **—**represents a significant correlation in T(+) men.

Figure 4 depicts the physiological relationships between the activity of ACS, DGAT, and CD36 content (per mg lipid) and the rate of FFA storage per kg abdominal and femoral adipose tissue. There was a significant correlation between rates of FFA storage and DGAT activity (ρ = 0.71, p = 0.02) and CD36 content (ρ = 0.64, p = 0.02) in the abdominal adipose tissue of T(+) men only. No significant correlations were found in T(−) men.

**Figure 5:**
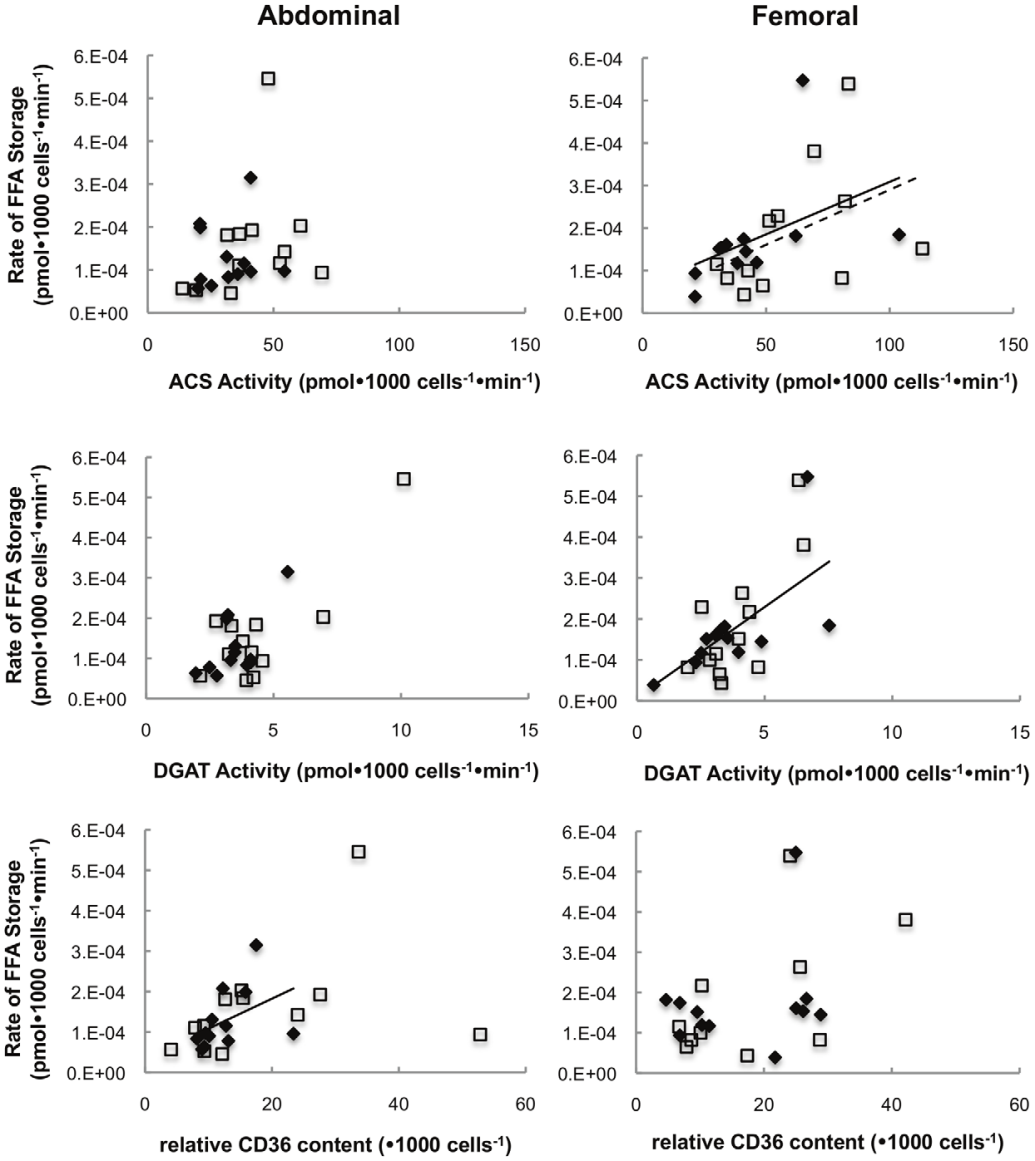
Cellular relationship between regional FA storage factors vs. regional FFA storage. Regional rates of FFA storage (pmol•1000 cells^−1^•min^−1^) vs. lipogenic protein activity expressed per 1000 cells. ♦represents T(+) men, □represents T(−) men, **—**represents a significant correlation in T(+) men, **- - -**represents a significant correlation in T(−) men.

When examining these relationships at the adipocyte level (per 1000 cells) we found that direct FFA storage rates in femoral adipose tissue were correlated (ρ = 0.62, p = 0.03) with femoral adipose tissue ACS activity in T(−) men (Figure 5). ACS (ρ = 0.73, p = 0.007) and DGAT activity (ρ = 0.71, p = 0.01) were correlated with FFA storage rates in femoral adipose tissue of T(+) men. In abdominal fat of T(+) men, FFA storage rates was correlated (ρ = 0.59, p = 0.04) with CD36 content.

#### Relationship between adipocyte FA storage factors

The relationships between adipocyte CD36, ACS and DGAT (per 1000 cells) were also examined. In abdominal fat, ACS and DGAT activities were correlated (ρ = 0.69, p = 0.01) in T(+) men. In T(−) men, abdominal ACS activity and CD36 content were correlated (ρ = 0.68, p = 0.01). In femoral adipose tissue only ACS and DGAT activity were correlated (ρ = 0.86, p<0.001) in T(+) men, whereas in T(−) men, ACS activity, DGAT activity, and CD36 content were correlated with each other (ACS vs. DGAT ρ = 0.65, p = 0.02; ACS vs. CD36 ρ = 0.76, p = 0.01; DGAT vs. CD36 ρ = 0.82, p = 0.004).

## Discussion

Androgens have remarkable effects on body fat distribution, yet the adipose tissue/cell specific effects of testosterone on FA metabolism remain somewhat of a mystery. In this study we measured adipose tissue FA storage in vivo together with adipocyte factors that regulate storage in men with chronic (>6 months) hypogonadism and a group of age and body composition-matched, eugonadal men. Hypogonadal men stored a greater proportion of both dietary fatty acids and FFA in lower body fat than did eugonadal men. Of note, hypogonadal men also had a selective, almost 2-fold increase in femoral adipocyte ACS activity, which could account for the alteration in FA storage. Whereas eugonadal men had significant regional differences (upper>lower body) in adipose tissue DGAT activity, hypogonadal men did not. At the cellular level, femoral ACS activity was correlated with direct adipose tissue FFA storage rates in both T(+) and T(−) men, but the greater adipose ACS activity in T(−) men appears to relate to greater FFA storage per cell (Figure 4, upper right panel). Our findings strongly suggest that testosterone regulates adipose tissue FA metabolism, at least in part through suppressing femoral adipocyte ACS activity.

In our study eugonadal and hypogonadal men were intentionally matched for general body composition. Despite this, T(+) men had abdominal fat cells that were ∼20% smaller and femoral fat cells that were ∼6% larger than T(−) men. Thus, for an equivalent amount of UBSQ fat T(+) men had almost 20% more adipocytes, but very similar numbers of LBSQ adipocytes per kg fat compared with T(−) men. This testosterone effect on adipocyte size explains how T(+) men could have similar DGAT activity per 1000 cells in abdominal and femoral fat (Table 4), yet have greater abdominal than femoral DGAT activity per tissue weight.

The effect of testosterone deficiency to disproportionately increase femoral adipocyte ACS could have negative consequences. The increased ACS both per tissue weight and per adipocyte in T(−) men without quantitatively similar changes in DGAT activity suggests a potential for an accumulation of intracellular acylated fatty acids. Acylated FA's are known to act as signaling molecules, modulating the activity of other enzymes involved in FA metabolism such as acyl CoA synthase, acetyl CoA carboxylase, AMP-activated kinase-kinase, HMG CoA reductase, carnitine palmitoyl transferase, and hormone sensitive lipase [34]. Although most (or all) of acylated FAs taken up by femoral adipocytes may be stored as triglyceride, our approach to measuring FA storage cannot distinguish triglycerides from other complex lipids. Thus, although we are confident that the proportion of meal FA and plasma FFA stored in lipids of LBSQ was greater in the T(−) compared to the T(+) men, we cannot exclude accumulation of fatty acids in compounds upstream of triglyceride.

In testosterone deficient men abdominal DGAT activity and CD36 content were not correlated with rates of postabsorptive FFA storage per unit fat tissue, suggesting these lipogenic proteins are not rate limiting under these circumstances. The strength of the correlations (ρ-values) between both DGAT activity and CD36 content were very different in the T(+) and T(−) groups, suggesting that testosterone may regulate one, but not both, of these proteins. However, a data point from one T(+) individual with abdominal DGAT activity >15 pmol•mg lipid^−1^•min^−1^ (see Figure 4) largely drove the significant relationship between DGAT activity and direct FFA storage rates. We carefully examined all data related to this participant and the experiments and found no reason to exclude this greater value. Moreover, absent this outlier there remained a significant correlation between DGAT and direct FFA storage in T(+) men.

The correlation of direct FFA storage in adipocytes of T(−) and T(+) men with ACS activity suggests that ACS may be a rate limiting step, although the apparent co-regulation of several FA storage factors makes it difficult to be confident that a single factor can explain our observations regarding fatty acid storage. If greater ACS activity is responsible for increased direct FFA storage, pre-ACS FFA availability must be sufficiently great that amplified ACS activity allows greater storage. We note that T(+) men had greater fat oxidation, and therefore perhaps less FA available for storage into adipose tissue, than T(−) men. It is possible FA that are not oxidized are stored preferentially in LBSQ of T(−) men.

Greater portions of both meal fatty acids and direct FFA storage were found in LBSQ of T(−) than T(+) men, which was in accordance with our predefined hypothesis relating to the effects of testosterone or regional fat storage. The caveat in interpreting these percentages is that they are a function of the amount of LBSQ present. The difference in the average LBSQ mass between group of ∼1 kg was not significant. Therefore, it is more likely that the differences observed are based on greater FFA storage rather than depot mass. There were no differences in regional LPL activity or the relationship between regional fed LPL activity and meal FA storage per g adipose tissue lipid between T(+) and T(−) men. This suggests that the differences in meal FA storage between eugonadal and hypogonadal men are not related to effects on adipose tissue LPL activity. Because no between group differences in LPL activity were observed, we did not measure tissue content of local factors, such as angiopoietin-related protein 4, which may regulate LPL.

A major strength of this study is that participants were matched for age, BMI and body composition, allowing us to isolate the effects of testosterone on adipose tissue metabolism. The results of our studies are generally consistent with previous reports that testosterone has effects to reduce adipose tissue LPL activity [5]–[7], enhance fat oxidation [35] and reduce abdominal adipocyte size [36]. Thus, the population we selected for these experiments appears to have faithfully reproduced findings from other groups. A potential limitation is that only men with prostate cancer remission are allowed to be medically hypogonadal for extended periods of time; otherwise men discovered to be hypogonadal receive testosterone replacement therapy. The men in our study had prostate cancer in complete remission, and were healthy as indicated via physician assessment and undetectable PSA. Another caveat regarding our results is that men in the T(−) group were both testosterone and estrogen deficient. Because estrogen concentrations in eugonadal men are normally <20% of those found in premenopausal women, we suspect the differences we observed were most likely attributable to testosterone. We could not find publications describing molecular regulation of adipocyte ACS or DGAT by testosterone or estrogen, which leaves us without a satisfying explanation for how sex steroids modulate fatty acid storage factors. Indeed, it is possible that testosterone suppresses lower body adipocyte ACS activity via indirect, rather than direct (gene regulation) pathways.

In summary, out data suggest that chronic testosterone deficiency in men alters the adipocyte enzymes involved in fatty acid storage. In testosterone sufficient men, DGAT seems to play a predominant role in overall fat storage. Testosterone deficiency in men increases femoral ACS activity, which is associated with the rate of FFA storage in the femoral depot. In agreement with our hypothesis, the proportion of direct and meal fat stored in femoral adipose tissue of T(−) men was greater than that in T(+) men. Our results provide a comprehensive examination of the chronic effects of testosterone deficiency on fatty acid metabolism, which we hope will lead to greater insights into the role of androgens in body fat distribution and adipose tissue metabolism in humans.
